# DBBM shows no signs of resorption under inflammatory conditions. An experimental study in the mouse calvaria

**DOI:** 10.1111/clr.13538

**Published:** 2019-09-30

**Authors:** Ulrike Kuchler, Gabriel Mulinari dos Santos, Patrick Heimel, Alexandra Stähli, Franz Josef Strauss, Stefan Tangl, Reinhard Gruber

**Affiliations:** ^1^ Department of Oral Surgery University Clinic of Dentistry Medical University of Vienna Vienna Austria; ^2^ Department of Oral Surgery and Integrated Clinic Universidade Estadual Paulista “Júlio de Mesquita Filho” Araçatuba Dental School Araçatuba Brazil; ^3^ Core Facility Hard Tissue and Biomaterial Research Karl Donath Laboratory University Clinic of Dentistry Medical University of Vienna Vienna Austria; ^4^ Department of Oral Biology University Clinic of Dentistry Medical University of Vienna Vienna Austria; ^5^ Ludwig Boltzmann Institute for Clinical and Experimental Traumatology Vienna Austria; ^6^ Austrian Cluster for Tissue Regeneration Vienna Austria; ^7^ Department of Periodontology School of Dental Medicine University of Bern Bern Switzerland; ^8^ Department of Conservative Dentistry School of Dentistry University of Chile Santiago Chile

**Keywords:** bone regeneration, bone substitutes, calvaria, inflammation, mice, osteoclasts

## Abstract

**Objectives:**

Deproteinized bovine bone mineral (DBBM) is not resorbable. However, the behavior of DBBM under inflammatory conditions remains unclear. Aim of the study was therefore to evaluate the resorption of DBBM under local inflammatory conditions in vivo using the calvarial osteolysis model.

**Methods:**

In thirty adult BALB/c mice, DBBM was implanted into the space between the elevated soft tissue and the calvarial bone. Inflammation was induced either by lipopolysaccharides (LPS) injection or by polyethylene particles (Ceridust) mixed with DBBM. Three modalities were randomly applied (*n* = 10 each): (a) DBBM alone (control), (b) DBBM + LPS, and (c) DBBM + polyethylene particles (Ceridust). Mice were euthanized on day fourteen, and each calvarium was subjected to histological and µCT analysis. Primary outcome was the size distribution of the DBBM particles. Secondary outcome was the surface erosion of the calvarial bone.

**Results:**

Histological and µCT analysis revealed that the size distribution and the volume of DBBM particles in the augmented site were similar between DBBM alone and the combinations with LPS or polyethylene particles. Moreover, histological evaluation showed no signs of erosions of DBBM particles under inflammatory conditions. µCT analysis and histology further revealed that LPS and the polyethylene particles, but not the DBBM alone, caused severe erosions of the calvarial bone as indicated by large voids representing the massive compensatory new immature woven bone formation on the endosteal surface.

**Conclusions:**

Local calvarial bone but not the DBBM particles undergo severe resorption and subsequent new bone formation under inflammatory conditions in a mouse model.

## 1 INTRODUCTION

Knowledge of bone biology in conjunction with implant placement has enormously increased in the last decades. However, surprisingly little is known about the dynamics of bone resorption. Chronic inflammation, being a hallmark of peri‐implantitis (Berglundh, Gislason, Lekholm, Sennerby, & Lindhe, 2004; Lindhe, Berglundh, Ericsson, Liljenberg, & Marinello, 1992) and periodontitis (Page, Engel, Narayanan, & Clagett, 1978), cause catabolic changes in bone and may lead to implant or tooth loss. Thus, we have solid evidence about the destructive impact of chronic inflammation on the resorption of alveolar bone in peri‐implantitis (Salvi, Cosgarea, & Sculean, 2017) and periodontitis (Bartold & Van Dyke, 2013). Unexpectedly, however, the impact of chronic inflammation either septic or aseptic on the resorption of bone substitutes, independent if they are of natural or synthetic origin, is sparse and almost exclusively based on histological observations (Scarano, Cholakis, & Piattelli, 2017). The question then arises of what happens to the bone substitutes under different inflammatory conditions? This lack of knowledge is unexpected because inflammation of an area previously augmented with bone substitutes, for example, an extraction socket (Kim et al., 2017), is rather common. Consequently, there is a clear demand to increase our knowledge on the performance of bone substitutes under inflammatory conditions.

Deproteinized bovine bone mineral (DBBM) is considered a slow resorbing bone substitute that is detectable after more than a decade in biopsies of augmented sites (Jensen, Bosshardt, Gruber, & Buser, 2014). Histology allows taking a detailed observation of the multinucleated cells that cover a part of the DBBM surface (Jensen, Gruber, Buser, & Bosshardt, 2015). The multinucleated cells express the tartrate‐resistant acid phosphatase and show a ruffled border with cytoplasmic extensions toward the mineralized surface (Jensen et al., 2015). What cannot be observed, however, is the hallmark of osteoclast function, Howship's lacunae (Chakar et al., 2014). This is not particularly surprising because biomaterials behave differently than autografts, also when osteoclasts are directly seeded on DBBM (Perrotti, Nicholls, Horton, & Piattelli, 2009; Taylor, Cuff, Leger, Morra, & Anderson, 2002). These in vitro studies support the concept that DBBM is resorbable, at least to a minor extent as compared to a bone surface under the same experimental conditions. The extent to which DBBM is resorbable under chronic inflammatory conditions in vivo remains unclear.

Our group discovered, using a minipig augmentation model, that when the occlusive protection of the augmented site is impaired, severe resorption of DBBM is initiated (Busenlechner et al., 2012). Osteoclasts form Howship's lacunae in the DBBM, and within 12 weeks, some augmented sites were almost completely resorbed (Busenlechner et al., 2012). Since then, the findings were not reproduced, likely because bone augmentation usually works predictably in vivo and clinicians accept that a certain percentage of bone resorption occurs. Nevertheless, our observations have led to speculations on the impact of the microenvironment on the resorption of DBBM and possibly of other biomaterials. There is ahypothesis that DBBM placed in an unstable ectopic soft tissue is subjected to resorption (Busenlechner et al., 2012; Buser et al., 2013). To gain a better understanding of the underlying cellular and molecular mechanisms, preclinical models were introduced.

Calvarial bone of rodents became an established model to study bone resorption induced by inflammation in vitro and in vivo (Kassem et al., 2015). Severe resorption of the calvarial bone can be induced by polyethylene particles (von Knoch et al., 2004; Nich et al., 2010) or by lipopolysaccharides (LPS) injections (Kassem et al., 2015). Osteoclastogenesis is rapidly initiated, and the resorption of the thin cortex of the calvarial bone can be measured by µCT and histology. The concept of using mouse calvarial bone for augmentation is not entirely new (Ohba et al., 2016), but we are the first to combine it with the calvarial osteolysis model to study the behavior of bone grafts, in this case DBBM, under inflammatory conditions. As this mouse calvarial model enables a simple access for both the surgical procedure and the LPS injection, it allows to verify our null hypothesis that DBBM shows no signs of resorption under inflammatory conditions. We report here that LPS and polyethylene particles cause severe catabolic changes in the calvarial bone, however, not being accompanied by substantial alterations of the size distribution of the DBBM particles.

## 2 MATERIAL AND METHODS

### 2.1 Study design

The Medical University of Vienna ethical review board for animal research approved the study protocol (GZ BMWFW‐66.009/0193‐WF/V/3b/2016‐2016). The study was performed in 2017 at the Department of Biomedical Research of the Medical University of Vienna in accordance with the ARRIVE guidelines. Thirty male BALB/c mice (8–10 weeks, 20–25 g) from the Division for Biomedical Research (Himberg, Austria) were randomly divided into three groups with 10 animals each based on a random number generator (GraphPad, La Jolla, CA). The animals were treated according to the guidelines for animal care with free access to water and a standard diet (Kilkenny, Browne, Cuthill, Emerson, & Altman, 2010).

### 2.2 Calvarial osteolysis model

UK and FJS performed the surgeries. All animals received ketamine 100 mg/kg (AniMedica) and xylazine hydrochloride 5 mg/kg (Bayer Austria) by intramuscular injection. An incision was made over the calvaria. The periosteum was elevated off the external cortex of the calvarium by sharp dissection, and three treatment modalities were randomly applied: (a) DBBM alone (50 mg, Bio‐Oss, Geistlich), (b) DBBM (25 mg) together with polyethylene particles (25 mg; 1%; Ceridust VP 3620; Clariant), and (c) alternatively, in the third group after the DBBM (50 mg) augmentation inflammation was induced by two local injection of LPS from *Escherichia coli* serotype O55: B5 (25 mg/kg, Sigma) at day 2 and day 5 next to sagittal suture directly into the augmented area. The wounds were closed in two layers with resorbable sutures (Vicryl 6–0; Ethicon GmbH). For pain relief, buprenorphine 0.06 mg/kg s.c. (Temgesic^®^, Temgesic, Reckitt and Colman Pharm.) and piritramide in drinking water ad lib were administered. Mice were euthanized on day fourteen with an overdose of sodium pentobarbital at 300 mg/kg i.p., and each calvarium was subjected to histological and micro‐computed tomographic (µCT) analysis.

### 2.3 Micro‐CT analysis

**Figure 1:**
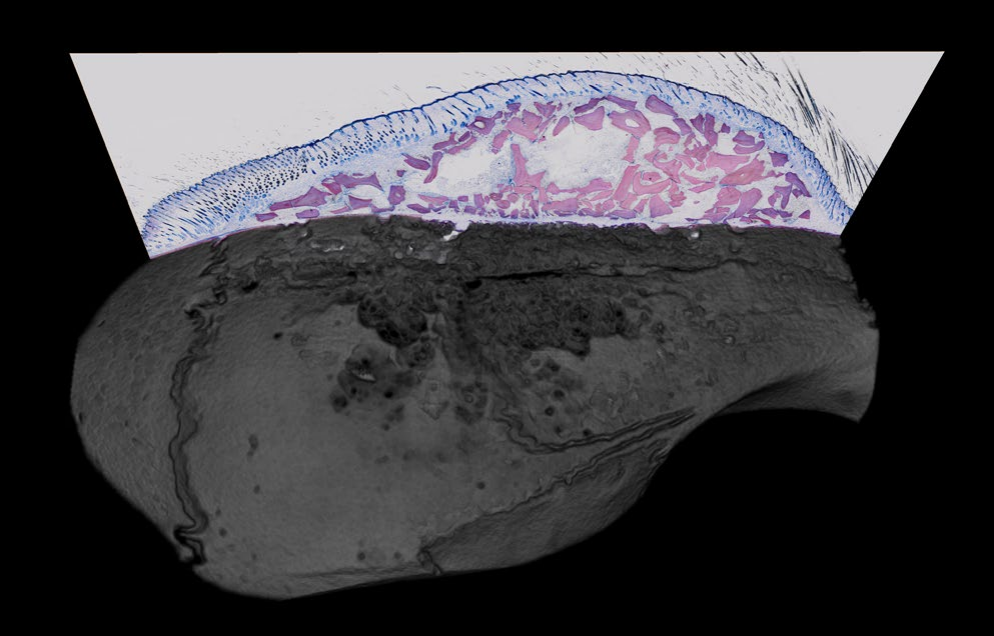
Deproteinized bovine bone mineral (DBBM) shows no signs of resorption under inflammatory conditions. Representative image of the surgical site combining µCT and histology. The severe erosions of the calvarial bone are visible while the morphology of the DBBM particles is not obviously disturbed

After euthanasia, the heads were fixed in phosphate‐buffered formalin (Roti‐Histofix 4%, Carl Roth). µCT scans were made using a SCANCO µCT 50 (SCANCO Medical AG) at 90 kV/200 µA with an isotropic resolution of 17.2 µm and an integration time of 500 ms. The images were rotated using Amira 6.2 (Thermo Fisher Scientific) so that the rostral axis lies along the *y*‐axis with the augmented area oriented toward slice 0 in the approximate center of the image. The images were then registered using rigid registration so that the calvaria are at the same position and orientation in all scans. Region of interest (ROI) was defined comprising the augmented compartments of the surgical site (Figure 1). Using the Definiens Developer XD2^®^ software (Version 2.1.1), the ROIs were positioned manually and automatically segmented from the µCT images with a specifically developed ruleset. At the interior surface of the calvarial bone, we determined the cortical porosity (void volume/tissue volume, Ct.Vd.V/TV in %), cortical bone volume/tissue volume (Ct.BV/TV in %), and cortical thickness (Ct.Th, mm). The DBBM particle size distribution was measured. In addition, the total DBBM graft volume (mm^3^) and the relative proportion of soft tissue in the augmented site (void volume/tissue volume, Vd.V/TV in %) were determined. Measurements and calculations were performed with the Definiens Developer XD2^®^ software (Version 2.1.1). Calibration and blinding of the examiner was not necessary as the software performed the analysis automatically and identically for all samples. For further µCT details and an overview on the scans, see Methods and Figure S1, respectively.

### 2.4 Histological analysis

All samples were dehydrated with ascending alcohol grades and embedded in light‐curing resin (Technovit 7200 VLC + BPO; Kulzer & Co.). Blocks were further processed using Exakt cutting and grinding equipment (Exakt Apparatebau). Thin‐ground sections from all samples were prepared according to Donath (Donath & Rohrer, 2003), in a plane parallel to the sagittal suture and through the center of the augmented area and stained with Levai–Laczko dye. The slices of around 20 µm were scanned using an Olympus BX61VS digital virtual microscopy system (DotSlide 2.4, Olympus) with a 20× objective resulting in a resolution of 0.32 µm per pixel and then evaluated. An overview of the augmented site is depicted in Figure 1.

### 2.5 Statistics

The data are presented graphically using scatterplots, overplotted by the mean and a corresponding bootstrap 95% confidence interval (CI). A non‐parametric approach was used for inference: In a first step, an ANOVA‐type permutation test was calculated based on B = 10000. In case of significance, pairwise post hoc permutation tests were performed, using the step‐down maxT procedure to account for multiple testing (Hothorn, Bühlmann, Dudoit, Molinaro, van der Laan, 2006, Westfall and Troendle 2008). All computations were done using R version 3.5.1 (R Core Team, 2012). Owing to the pilot nature of the study, the sample size was chosen based on experience from previous studies (Ohba et al., 2016) to balance the ability to measure significant differences while reducing the number of animals used.

## 3 RESULTS

### 3.1 Catabolic changes of the mouse calvarial bone by LPS and Ceridust—µCT data

**Figure 2:**
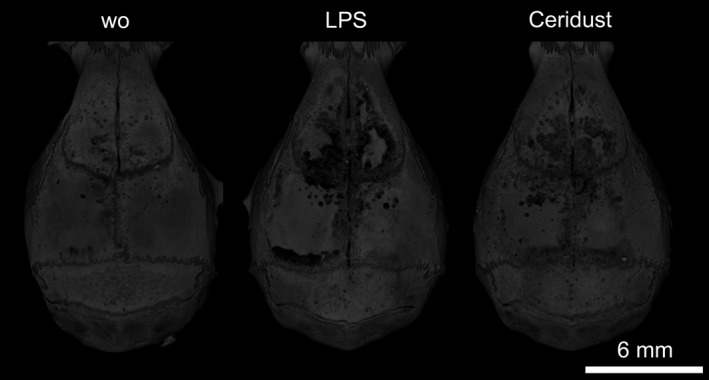
Lipopolysaccharides (LPS) and Ceridust induce calvarial bone resorption. µCT overview of the augmentation site. Periosteum was elevated, and the calvarial bone was augmented with deproteinized bovine bone mineral (DBBM). Inflammation was induced by local injection of lipopolysaccharides (LPS) from *Escherichia coli* serotype O55: B5 or Ceridust polyethylene particles. Note the severe erosions of the calvarial bone in the LPS and Ceridust group while only a few signs of resorption are visible on the calvarial bone augmented with DBBM alone

**Figure 3:**
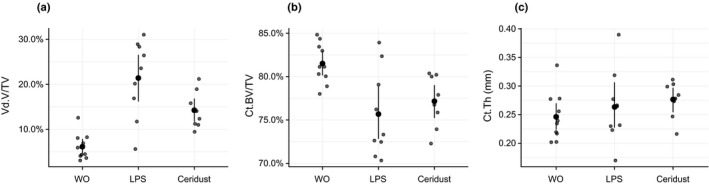
Lipopolysaccharides (LPS) and Ceridust alter the cortical porosity and cortical bone volume. Based on µCT data, cortical porosity (void volume/tissue volume, Vd.V/TV in %), cortical bone volume/tissue volume (Ct.BV/TV in %), and cortical thickness (Ct.Th in mm) were determined. Lipopolysaccharides (LPS) and Ceridust caused an increase of the Vd.S/TS with *p* < .001 and *p* = .062, respectively. LPS and Ceridust decreased cortical bone volume (Ct.BV/TV) with *p* = .006 and *p* = .062, respectively. Thickness of the cortical bone was not different between the three groups (*p* = .641). The data are presented using scatterplots with mean and a corresponding bootstrap 95% confidence interval

A total of three mice were lost during surgery, and as a result, a total of 27 mice were analyzed. From these 27 mice, seven showed flap dehiscences; three mice in the LPS group, two mice in the Ceridust group and two mice in control group (DBBM alone) without significant differences between the groups (*p* = .8011; Table S1). µCT analysis revealed that LPS and Ceridust caused catabolic changes in the form of large voids of the calvarial bone (Figure 2). The large voids represent the massive compensatory new immature woven bone formation on the endosteal surface. Quantitative analysis showed that in the control group (DBBM alone), the void volume in the cortical bone was 5.2% (CI: 4.1–7.8). Injection of LPS caused an almost fourfold increase of the mean void volume to 21.4% (CI: 16.9–28.3, *p* < .001). Ceridust also increased the mean void volume around twofold over controls to 14.2% (CI: 11.2–16.6, *p* = .062; Figure 3a). In support of these observations, there was an indirect‐proportional decrease of cortical bone volume (BV/TV) from 81.5% (CI: 80.1–83.3) in the control group to 75.7% (CI: 72.5–79.1, *p* = .006) in the LPS group and 77.2% (CI: 75.4–80.1, *p* = .062) in the Ceridust group, respectively (Figure 3b). The thickness of the cortical bone (Figure 3c) was not significantly changed by LPS or Ceridust (*p* = .641). Taken together, LPS and Ceridust caused severe erosion of the frontal calvarial bone indicated by the compensatory increase of new immature bone.

### 3.2 Catabolic changes of the mouse calvarial bone by LPS and Ceridust—histological data

**Figure 4:**
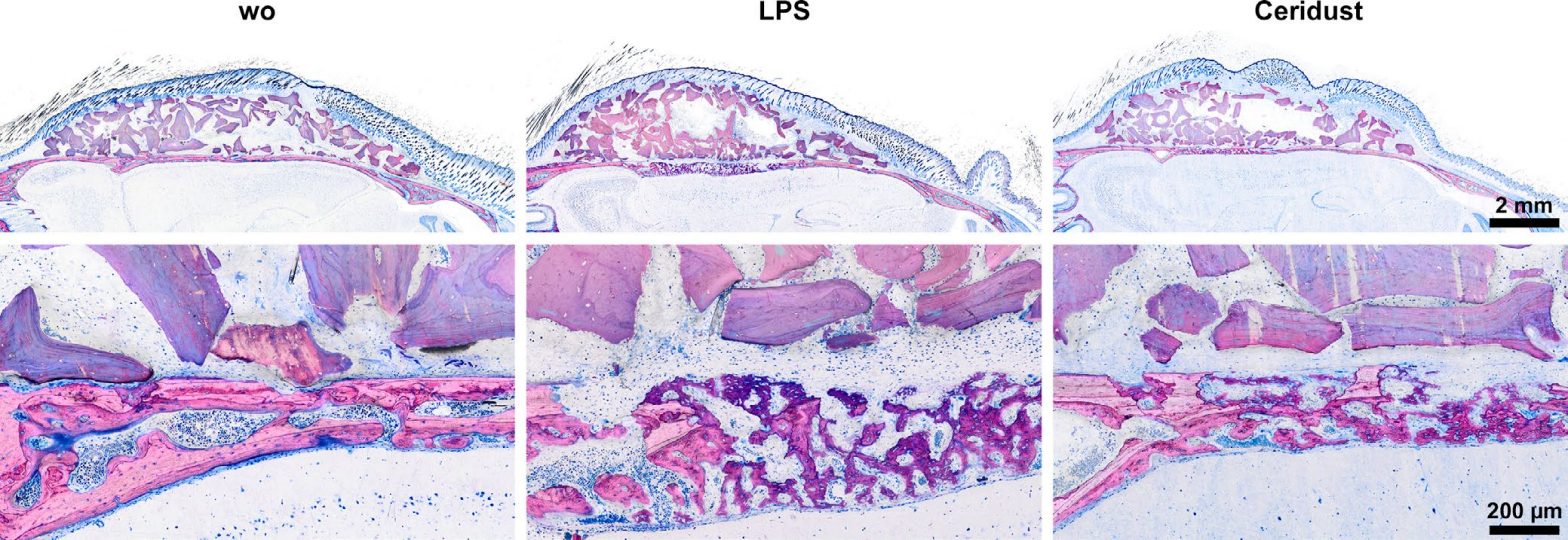
Bone resorption induced by Lipopolysaccharides (LPS) and Ceridust induce a compensatory repair. Ground sections of the augmented area stained with Levai–Laczko dye. The high magnification shows the transition of the calvarial bone and the DBBM particles with the strong signs of compensatory repair in the LPS and the Ceridust group supporting the successful initiation of inflammation. This process explains the significant increase Vd.S/TS and Ct.BV/TV in the LPS group. No signs of resorption are visible on the surface of deproteinized bovine bone mineral (DBBM) particles

Histological observations support the findings from the μCT analysis. As indicated in Figure 4a, the control group showed only few and small resorption sites and moderate new bone formation (Figure 4a). LPS and Ceridust caused extensive resorption as well as heavily compensatory endocranial bone formation underneath the cortical layer (Figure 4b,c). Resorption was strongest for Ceridust exhibiting more and larger resorption sites penetrating the cortical bone. Resorption often affected half of the parietal bone. Compensatory bone formation resulted in a loose network of immature woven bone with a high void volume, as indicated by the µCT analysis.

### 3.3 No changes of the DBBM particles by LPS and Ceridust—µCT

**Figure 5:**
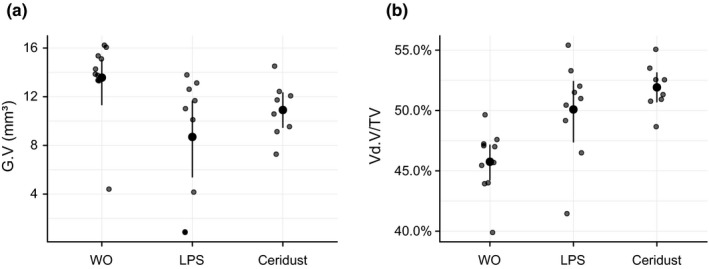
No changes of the deproteinized bovine bone mineral (DBBM) particles by lipopolysaccharides (LPS) and Ceridust. Based on µCT data, different regions of interest (ROIs) were segmented. We determined the total graft volume (G.V in mm^3^) and the porosity of the augmented area (void volume/tissue volume, Vd.V/TV in %). The data are presented using scatterplots with mean and a corresponding bootstrap 95% confidence interval

**Figure 6:**
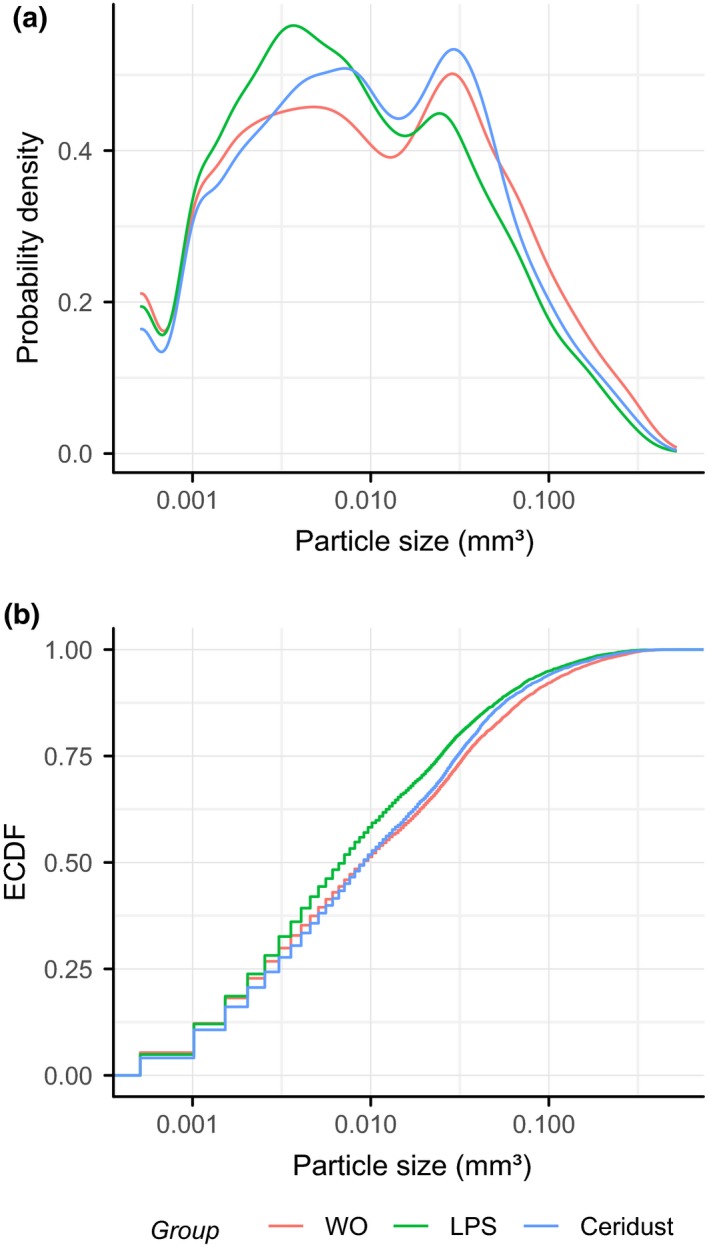
No changes in the particle size of the deproteinized bovine bone mineral (DBBM) by lipopolysaccharides (LPS) and Ceridust. Empirical cumulative probability functions for particle size (log scale). Statistical analysis showed no differences in the size distribution of the DBBM particles in controls compared with the LPS and Ceridust group

µCT analysis showed a relative DBBM volume of 13.5% (CI: 13.4–15.2) in the control group. No significant differences in DBBM volume were observed with LPS and Ceridust with 8.7% (CI: 4.1–12.6) and 10.9% (CI: 9.4–12.1), respectively (*p* = .057). The spaces between the DBBM particles or void volume/TV in the augmented site were 45.8% (CI: 44.4–47.2) in the control group. Injection of LPS and the use of Ceridust caused no significant changes in the volume of DBBM G.V/TV with 50.1% (CI: 49.2–52.0) and 51.9% (CI: 50.9–52.8), respectively (*p* = .092; Figure 5a). However, there is at least a trend that inflammation causes looser distribution of the DBBM particles in the augmented site compared to a control situation. We next determined whether LPS and Ceridust changes the size distribution of the DBBM particles in the augmented site. The size distribution of the DBBM particles ranged from 0.001 mm^3^ to more than 0.1 mm^3^ (Figure S2), but showed no significant differences when controls were compared to LPS and Ceridust groups (Figure 6).

### 3.4 No changes of the DBBM particles by LPS and Ceridust—histological analysis

**Figure 7:**
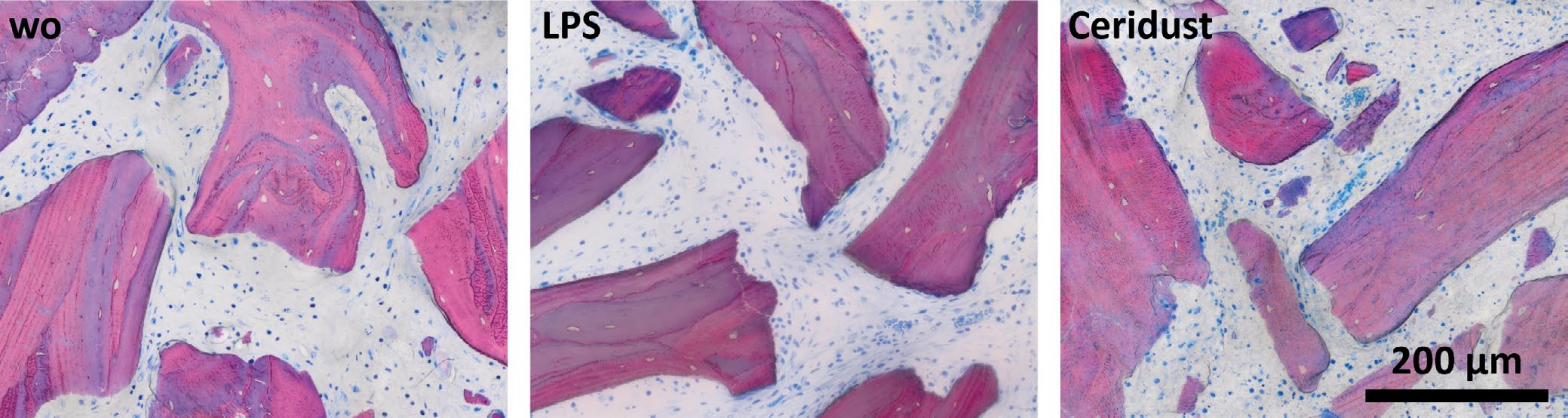
Deproteinized bovine bone mineral (DBBM) shows no signs of resorption irrespective of inflammatory condition. The high magnification shows the DBBM particles in controls and in the inflammatory LPS and Ceridust group. No signs of resorption are visible on the surface of DBBM particles independent of the inflammation induced, by LPS or Ceridust

Histology supports these observations as there was no obvious increase in the presence of macrophages or osteoclast‐like cells caused by LPS and Ceridust on the surface of DBBM (Figure 7). In all treatment groups, dehiscence occurred, yet, DBBM was encased between the skin of the scalp and the bones of the skull. In a large number of cases among all three groups, the skin overlying the bone substitute material showed dehiscence of considerable size. Histological signs of inflammation were strongest in the vicinity of the skin defects. In the augmented areas, inflammatory signs were weak and showed no considerable differences between the groups. The size of DBBM particles seemed to be undiminished. Furthermore, particle size and distribution appeared similar in all treatment groups (Figure 7). Taken together, even though DBBM treated with LPS or Ceridust causes a resorption of calvarial bone and a subsequent formation of new woven bone, no obvious changes of DBBM itself occurred between the treatment groups.

## 4 DISCUSSION

The results presented herein demonstrate that LPS and Ceridust cause a combination of intense resorption and a following formational activity in the mouse calvaria bone. The size distribution and the overall volume of DBBM particles were, however, unaffected by LPS and Ceridust. We therefore conclude that the inflammatory response to LPS and Ceridust that causes severe resorption of the calvarial bone did not result in the catabolic changes of DBBM particles. This observation is relevant considering that it provides insight into the behaviour of DBBM under inflammatory conditions. The occurrence of such an inflammatory scenario cannot be ruled out in a clinical setting, for example, when socket preservation in a patient affected by periodontitis is performed.

The severe catabolic changes of calvarial bone described here upon exposure to LPS or Ceridust are consistent with earlier reports (Kassem et al., 2015) that used LPS injections or polyethylene particles. However, in these earlier studies, LPS from P. gingivalis and the TLR2 agonist Pam2 were injected over the calvaria without augmentation for only 6 days and no analysis of bone porosity was performed (Kassem et al., 2015; von Knoch et al., 2004). We have used LPS from *E. coli* serotype O55: B5 known to activate TLR4 signaling, which is the major pathway inflammatory osteolysis in mouse models (Gruber, 2019). We have modified the protocol of Nich et al. (Nich et al., 2010) who distributed 20 µg Ceridust VP 3620 over the intact periosteum using a sterile sharp surgical spoon. After 14 days postoperatively, quantitative µCT imaging revealed an approximately 30% change of osteolysis that is based on TNF signaling (Merkel et al., 1999). Therefore, our µCT and histological data support previous observations that LPS and Ceridust can provoke inflammation‐induced changes of the calvarial bone. This is the first study to show this catabolic response in the presence of DBBM in a mouse calvarial augmentation model.

In line with previous research, we found sites of bone resorption by histological analysis that correspond to the sites of high void volume shown in the µCT analysis. .LPS and Ceridust triggered the formation and activity of osteoclasts leading to the erosion of the bone indicated by a compensatory woven bone formation and high void volume. Perhaps more important, we can confirm that the specimens with a high void volume are identical with those showing compensatory immature woven bone in the histology. Our observation that µCT and histology are congruent is a solid proof that the automated segmentation used for the structural analysis provided valid information. The present study is thus not only new with respect to the preclinical augmentation and inflammation model, but also because we present an innovative approach to quantitatively assess calvarial osteolysis with ensuing bone formation induced by LPS and Ceridust. We therefore provide evidence that in the mouse calvaria augmentation model DBBM is preserved under transient inflammatory conditions.

An earlier study by our group suggested that DBBM is resorbable in a pig calvaria augmentation model under as yet unexplained conditions (Busenlechner et al., 2012). It is possible that an inflammatory environment caused the underlying molecular and cellular mechanisms leading to resorption of DBBM. Resorption of DBBM as part of the remodeling process was earlier suggested (Zitzmann, Scharer, Marinello, Schupbach, & Berglundh, 2001). We have determined the DBBM volume and also analyzed the size distribution of all three groups of DBBM particles—the control, LPS, and Ceridust group. Independent of the severe inflammatory reaction, we found no statistical changes in the DBBM volume. Nevertheless, there was a trend that inflammation causes a looser distribution of DBBM particles in the augmented site, likely due to an edema and not osteoclast activity. More relevant are our findings that the characteristic size distribution of DBBM in the mouse calvaria with the majority of particles ranging between 0.001 mm^3^ to more than 0.1 mm^3^ was not considerably changed in the presence of LPS and Ceridust. These observations together with the evidence from histology suggest that DBBM particles are not undergoing resorption.

However, our mouse augmentation model has limitations as is does not represent long‐term chronic inflammation and also DBBM in the soft tissue of the oral mucosa may behave differently than in the calvaria sites. The present model represents an acute and a transient inflammation indicated by a compensation of the severe osteolysis through an increase in the osteoblast activity that would otherwise be suppressed during ongoing inflammation (Diarra et al., 2007). Apart from the clear signs of former osteolysis and the soft tissue dehiscence, no signs of an ongoing inflammation were noticed. It can be suggested that at the time of tissue harvesting, the inflammation had ceased and regeneration took over. This is also why the number of inflammatory cells could not be quantified. The present model does not reflect the resorption pattern that has been observed using other animal models (Busenlechner et al., 2012; Gruber, 2019) or in clinical situations of chronic inflammation and inflammatory osteolysis. Consequently, the effects of chronic inflammation on DBBM need to be further evaluated. Moreover, wound dehiscence, as was seen in all groups, is unpredictable and might result in infection, graft loss, or bone resorption. Since it was observed in all groups, it cannot be attributed to the higher amount of calvarial bone erosion in the LPS and Ceridust group. Another limitation is that the particles were stabilized only by the elevated periosteum; therefore, we cannot rule out that micro‐movements might have displaced the biomaterial affecting the  graft consolidation. However, the primary endpoint was related to the possible graft resorption under inflammatory conditions and in this sense the histology support our findings. There is thus a demand to study the behaviour of DBBM in larger preclinical models under chronic inflammatory conditions. 

Taken together, the findings presented here support the idea that short‐time inflammation per se is not a key trigger for cellular degradation or resorption of DBBM particles.

## CONFLICT OF INTEREST

All authors disclose any potential sources of conflict of interest.

## AUTHOR CONTRIBUTIONS

UK and RG developed the hypothesis; UK, FJS, and AS conducted the surgery and collected the data; UK, GM, PH, RG, and ST analyzed the data; and RG and UK led the writing, and all authors supported writing of the manuscript.

## References

[clr13538-bib-0001] Bartold, P. M. , & Van Dyke, T. E. (2013). Periodontitis: A host‐mediated disruption of microbial homeostasis. Unlearning learned concepts. Periodontology 2000, 62(1), 203–217. 10.1111/j.1600-0757.2012.00450.x 23574467 PMC3692012

[clr13538-bib-0002] Berglundh, T. , Gislason, O. , Lekholm, U. , Sennerby, L. , & Lindhe, J. (2004). Histopathological observations of human periimplantitis lesions. Journal of Clinical Periodontology, 31(5), 341–347. 10.1111/j.1600-051X.2004.00486.x 15086615

[clr13538-bib-0003] Busenlechner, D. , Tangl, S. , Arnhart, C. , Redl, H. , Schuh, C. , Watzek, G. , & Gruber, R. (2012). Resorption of deproteinized bovine bone mineral in a porcine calvaria augmentation model. Clinical Oral Implants Research, 23(1), 95–99. 10.1111/j.1600-0501.2011.02198.x 21554404

[clr13538-bib-0004] Buser, D. , Chappuis, V. , Kuchler, U. , Bornstein, M. M. , Wittneben, J. G. , Buser, R. , … Belser, U. C. (2013). Long‐term stability of early implant placement with contour augmentation. Journal of Dental Research, 92(12 Suppl), 176S–182S. 10.1177/0022034513504949 24158332 PMC3860062

[clr13538-bib-0005] Chakar, C. , Naaman, N. , Soffer, E. , Cohen, N. , El Osta, N. , Petite, H. , & Anagnostou, F. (2014). Bone formation with deproteinized bovine bone mineral or biphasic calcium phosphate in the presence of autologous platelet lysate: Comparative investigation in rabbit. International Journal of Biomaterials, 2014, 367265 10.1155/2014/367265 24982676 PMC4058493

[clr13538-bib-0006] Diarra, D. , Stolina, M. , Polzer, K. , Zwerina, J. , Ominsky, M. S. , Dwyer, D. , … Schett, G. (2007). Dickkopf‐1 is a master regulator of joint remodeling. Nature Medicine, 13(2), 156–163. 10.1038/nm1538 17237793

[clr13538-bib-0007] Donath, K. , & Rohrer, M. (2003). Bone sectioning using the exakt system In An Y. H., & Martin K. L. (Eds.), Handbook of histology methods for bone and cartilage. Totowa, NJ: Humana Press.

[clr13538-bib-0008] Gruber, R. (2019). Osteoimmunology: Inflammatory osteolysis and regeneration of the alveolar bone. Journal of Clinical Periodontology, 46(Suppl 21), 52–69. 10.1111/jcpe.13056 30623453

[clr13538-bib-0009] Hothorn, T. , Bühlmann, P. , Dudoit, S. , Molinaro, A. , & van der Laan, M. J. (2006). Survival ensembles. Biostatistics., 7(3), 355–73. 16344280 10.1093/biostatistics/kxj011

[clr13538-bib-0010] Jensen, S. S. , Bosshardt, D. D. , Gruber, R. , & Buser, D. (2014). Long‐term stability of contour augmentation in the esthetic zone: Histologic and histomorphometric evaluation of 12 human biopsies 14 to 80 months after augmentation. Journal of Periodontology, 85(11), 1549–1556. 10.1902/jop.2014.140182 25008214

[clr13538-bib-0011] Jensen, S. S. , Gruber, R. , Buser, D. , & Bosshardt, D. D. (2015). Osteoclast‐like cells on deproteinized bovine bone mineral and biphasic calcium phosphate: Light and transmission electron microscopical observations. Clinical Oral Implants Research, 26(8), 859–864. 10.1111/clr.12376 24665833

[clr13538-bib-0012] Kassem, A. , Henning, P. , Lundberg, P. , Souza, P. P. , Lindholm, C. , & Lerner, U. H. (2015). Porphyromonas gingivalis stimulates bone resorption by enhancing RANKL (Receptor Activator of NF‐kappaB Ligand) through Activation of Toll‐like Receptor 2 in Osteoblasts. Journal of Biological Chemistry, 290(33), 20147–20158. 10.1074/jbc.M115.655787 26085099 PMC4536425

[clr13538-bib-0013] Kilkenny, C. , Browne, W. J. , Cuthill, I. C. , Emerson, M. , & Altman, D. G. (2010). Improving bioscience research reporting: The ARRIVE guidelines for reporting animal research. PLoS Biology, 8(6), e1000412 10.1371/journal.pbio.1000412 20613859 PMC2893951

[clr13538-bib-0014] Kim, J. J. , Schwarz, F. , Song, H. Y. , Choi, Y. , Kang, K. R. , & Koo, K. T. (2017). Ridge preservation of extraction sockets with chronic pathology using Bio‐Oss((R)) Collagen with or without collagen membrane: An experimental study in dogs. Clinical Oral Implants Research, 28(6), 727–733. 10.1111/clr.12870 27194177

[clr13538-bib-0015] Lindhe, J. , Berglundh, T. , Ericsson, I. , Liljenberg, B. , & Marinello, C. (1992). Experimental breakdown of peri‐implant and periodontal tissues. A study in the beagle dog. Clinical Oral Implants Research, 3(1), 9–16. 10.1034/j.1600-0501.1992.030102.x 1420727

[clr13538-bib-0016] Merkel, K. D. , Erdmann, J. M. , McHugh, K. P. , Abu‐Amer, Y. , Ross, F. P. , & Teitelbaum, S. L. (1999). Tumor necrosis factor‐alpha mediates orthopedic implant osteolysis. American Journal of Pathology, 154(1), 203–210. 9916934 10.1016/s0002-9440(10)65266-2 PMC1853441

[clr13538-bib-0017] Nich, C. , Marchadier, A. , Sedel, L. , Petite, H. , Vidal, C. , & Hamadouche, M. (2010). Decrease in particle‐induced osteolysis in ovariectomized mice. Journal of Orthopaedic Research, 28(2), 178–183. 10.1002/jor.20987 19725120

[clr13538-bib-0018] Ohba, S. , Sumita, Y. , Umebayashi, M. , Yoshimura, H. , Yoshida, H. , Matsuda, S. , … Sano, K. (2016). Onlay bone augmentation on mouse calvarial bone using a hydroxyapatite/collagen composite material with total blood or platelet‐rich plasma. Archives of Oral Biology, 61, 23–27. 10.1016/j.archoralbio.2015.10.012 26492524

[clr13538-bib-0019] Page, R. C. , Engel, L. D. , Narayanan, A. S. , & Clagett, J. A. (1978). Chronic inflammatory gingival and periodontal disease. The Journal of the American Medical Association, 240(6), 545–550. 10.1001/jama.1978.03290060047012 678330

[clr13538-bib-0020] Perrotti, V. , Nicholls, B. M. , Horton, M. A. , & Piattelli, A. (2009). Human osteoclast formation and activity on a xenogenous bone mineral. Journal of Biomedical Materials Research Part A, 90(1), 238–246. 10.1002/jbm.a.32079 18496862

[clr13538-bib-0021] R Core Team . (2012). R: a language and environment for statistical computing. Vienna: R Foundation for Statistical Computing ISBN: 3-900051-07-0. http://www.R-project.org

[clr13538-bib-0022] Salvi, G. E. , Cosgarea, R. , & Sculean, A. (2017). Prevalence and mechanisms of peri‐implant diseases. Journal of Dental Research, 96(1), 31–37. 10.1177/0022034516667484 27680028

[clr13538-bib-0023] Scarano, A. , Cholakis, A. K. , & Piattelli, A. (2017). Histologic evaluation of sinus grafting materials after peri‐implantitis‐induced failure: A case series. International Journal of Oral and Maxillofacial Implants, 32(2), e69–e75. 10.11607/jomi.5303 28291856

[clr13538-bib-0024] Taylor, J. C. , Cuff, S. E. , Leger, J. P. , Morra, A. , & Anderson, G. I. (2002). In vitro osteoclast resorption of bone substitute biomaterials used for implant site augmentation: A pilot study. International Journal of Oral and Maxillofacial Implants, 17(3), 321–330. 12074446

[clr13538-bib-0025] von Knoch, M. , Jewison, D. E. , Sibonga, J. D. , Turner, R. T. , Morrey, B. F. , Loer, F. , … Scully, S. P. (2004). Decrease in particle‐induced osteolysis in obese (ob/ob) mice. Biomaterials, 25(19), 4675–4681. 10.1016/j.biomaterials.2004.02.069 15120513

[clr13538-bib-0026] Westfall, P. H. , & Troendle, J. F. (2008). Multiple testing with minimal assumptions. Biometrical Journal, 50(5), 745–55. 10.1002/bimj.200710456 18932134 PMC3117234

[clr13538-bib-0027] Zitzmann, N. U. , Scharer, P. , Marinello, C. P. , Schupbach, P. , & Berglundh, T. (2001). Alveolar ridge augmentation with Bio‐Oss: A histologic study in humans. The International Journal of Periodontics & Restorative Dentistry, 21(3), 288–295. 11490406

