# Supplementary material for: DBBM shows no signs of resorption under inflammatory conditions. An experimental study in the mouse calvaria
Source: Clin Oral Implants Res. 2019 Sep 30;31(1):10–7. doi: 10.1111/clr.13538 (PMC7003744; doi:10.1111/clr.13538)
Supplement: Supplementary file 1 [file CLR-31-10-s001.docx]

**Appendices**

Supplement Figure 1 *Overview on the calvaria bone of the three groups* and Supplement Figure 2 *Size distribution and visualization of DBBM particles* appear at the end of the manuscript*.*


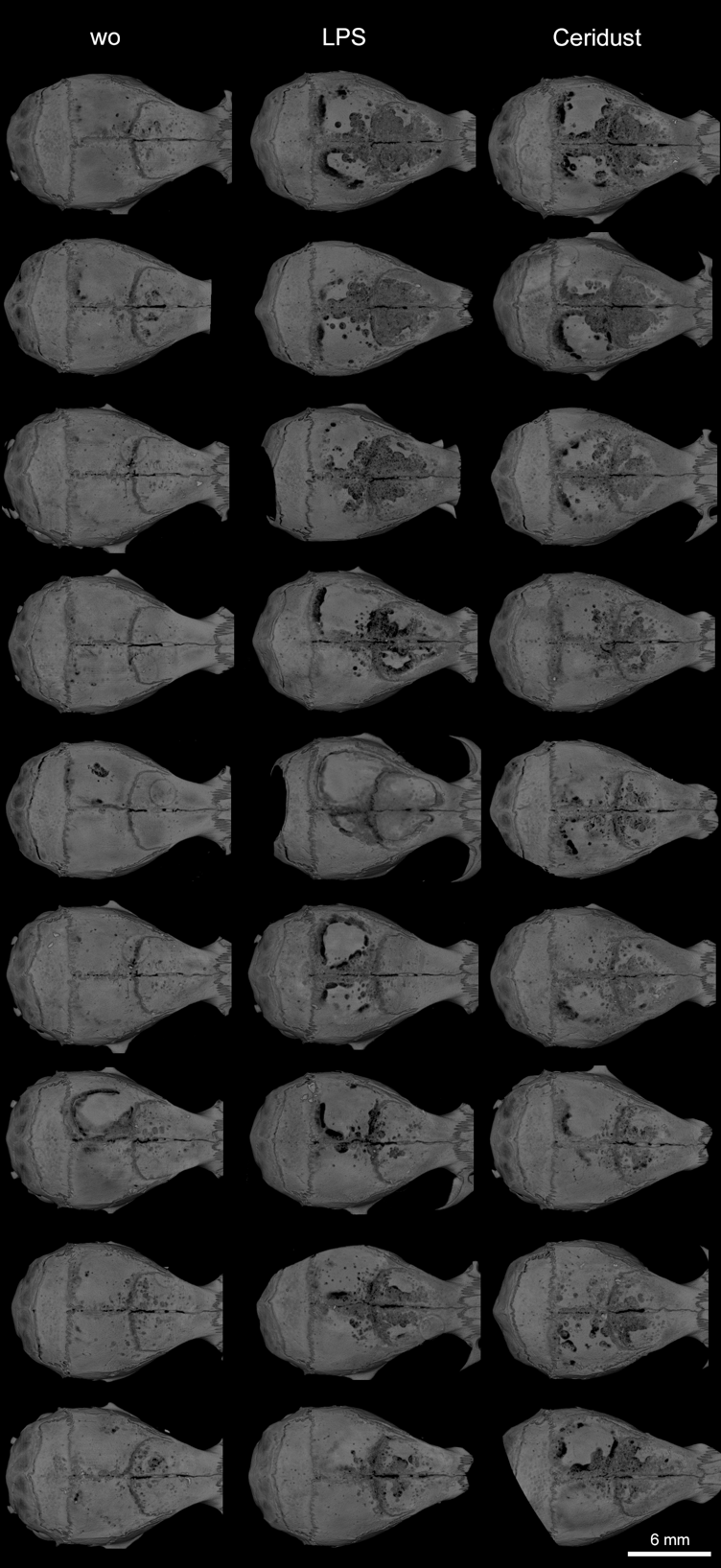


*Supplement Figure 1 Overview on the calvaria bone of the three groups*

Inflammation was induced by local injection of LPS from E. coli serotype O55: B5 or Ceridust polyethylene particles. Note the severe erosions of the calvarial bone in the LPS and Ceridust group.


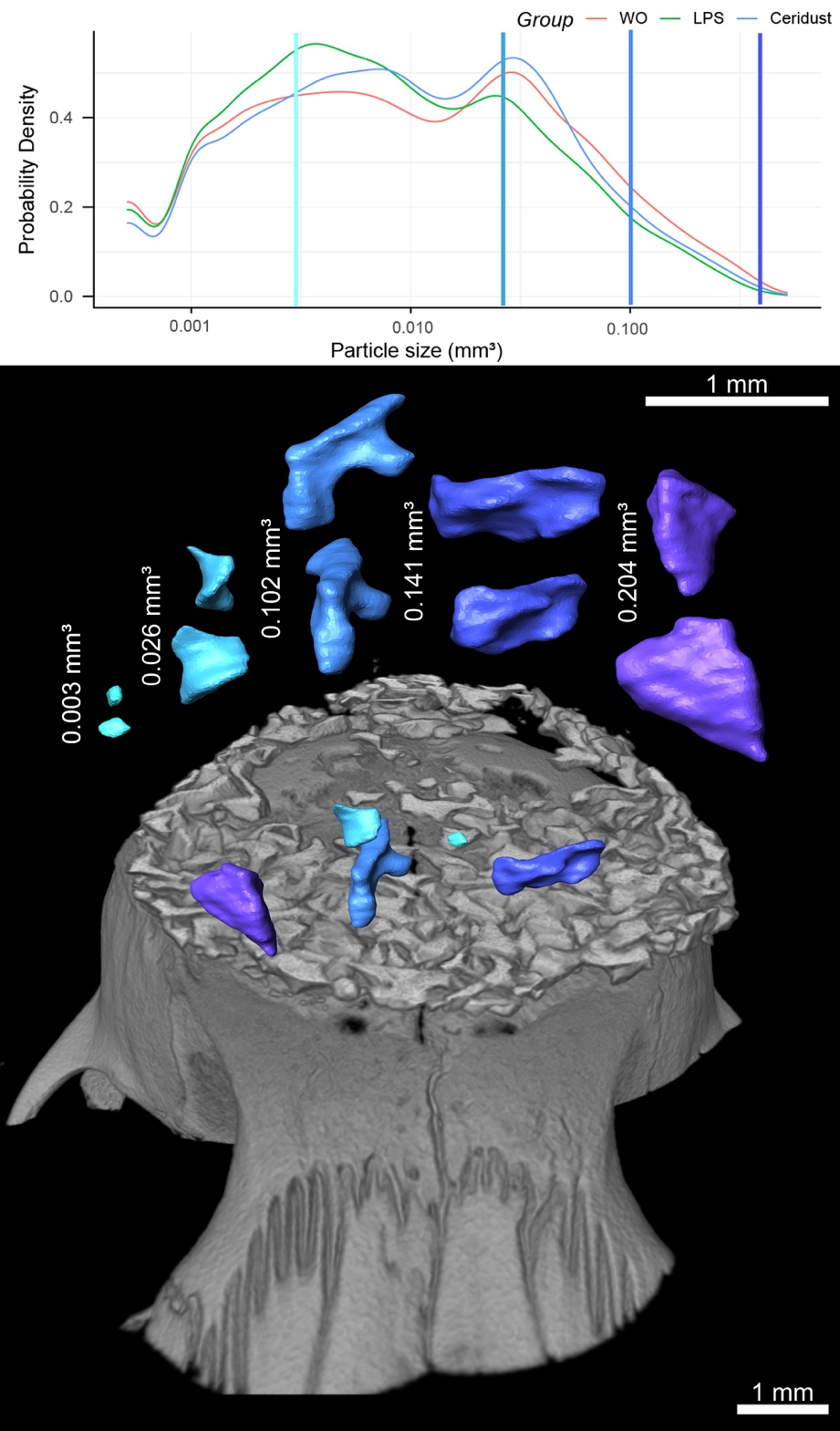


*Supplement Figure 2 Size distribution and visualization of DBBM particles*

µCT allowed segmentation of DBBM particles and consequently the evaluation of their size distribution in controls and in the LPS and Ceridust groups. Shown are representative examples of small and larger DBBM particles.
