# Supplementary material for: DBBM shows no signs of resorption under inflammatory conditions. An experimental study in the mouse calvaria
Source: Clin Oral Implants Res. 2019 Sep 30;31(1):10–7. doi: 10.1111/clr.13538 (PMC7003744; doi:10.1111/clr.13538)
Supplement: Supplementary file 2 [file CLR-31-10-s002.docx]

|  | Wound Dehiscence | | Total |
| --- | --- | --- | --- |
|  | Yes | No |  |
| WO | 2  *2.59*  (0.14) | 8  *7.41*  (0.05) | 10 |
| LPS | 3  *2.33*  (0.19) | 6  *6.67*  (0.07) | 9 |
| Ceridust | 2  *2.07*  (0.00) | 6  *5.93*  (0.00) | 8 |
|  | 7 | 20 | 27 |

Supplement Table 1. χ^2^ Calculations for wound dehiscence

χ^2^  =  0.444,     df  =  2,     χ^2^/df  =  0.22 ,         P(χ^2^ > 0.444)  =  0.8011

Expected values are displayed in *italics* and individual χ^2^values are displayed in parentheses.
