# Supplementary material for: DBBM shows no signs of resorption under inflammatory conditions. An experimental study in the mouse calvaria
Source: Clin Oral Implants Res. 2019 Sep 30;31(1):10–7. doi: 10.1111/clr.13538 (PMC7003744; doi:10.1111/clr.13538)
Supplement: Supplementary file 3 [file CLR-31-10-s003.docx]

**Supplement methods**

*Micro CT analysis*

The automatic segmentation was performed in several steps. The rough position of individual structures was found on a down sampled copy of the full images. The inner bone surface of the calvaria was found using region growing from a seed inside the skull constrained by surface tension. This way, the void volume inside the skull is found. The interior surface of the calvaria bone is located where this volume has contact to bone within the regions of interest. The bone of the calvaria was segmented by growing from the found surface in the dorsal direction constrained by a surface tension and appropriate thresholds for the intensity of the bone. Growth into DBBM particles in direct contact with the bone was prevented by surface tension and an intensity threshold. The DBBM graft volume was found using thresholding of the particles dorsal of the exterior bone surface of the calvaria followed by region growing constrained by a surface tension to fill up the void volume between the individual DBBM particles.

These regions (DBBM graft volume, calvaria bone, interior bone surface) where then copied to the full resolution images. The regions were adjusted to correct for the inaccuracy of the lower resolution using region growing with a combination of threshold and surface tension constraints. They were then thresholded to separate the tissues contained within each region (DBBM particles and void in the DBBM graft volume; bone and void in the calvaria bone and bone surface). Individual DBBM particles were separated using region growing. Before separation, most particles are connected with other particles in large objects. These objects were shrunk applying surface tension constraints so that sharper edges are removed more quickly than flat surfaces. The shrinkage results in a split between individual particles at the point of connection. Shrinkage of individual particles was stopped once a minimal size was reached. After this step, there is a single object in the approximate center of each DBBM particle. These objects are then grown constrained by thresholds and surface tension to once again fill their individual DBBM particle without merging with neighboring particles.
